# Can tranexamic acid in irrigation fluid reduce blood loss during monopolar transurethral resection of the prostate? A randomised controlled trial

**DOI:** 10.1080/2090598X.2022.2026011

**Published:** 2022-01-23

**Authors:** Ahmed Tawfick, Waleed Mousa, Ahmed Fawaz El-Zhary, Ahmed Mohamed Saafan

**Affiliations:** Urology Department, Faculty of Medicine, Ain Shams University, Cairo, Egypt

**Keywords:** Benign prostatic hyperplasia, haemorrhage, tranexamic acid, transurethral resection of prostate

## Abstract

**Objective:**

To assess the efficacity and safety of using tranexamic acid (TXA) in the irrigation solution during transurethral resection of the prostate (TURP).

**Patients and Methods:**

A total of 50 patients undergoing TURP for benign prostatic hyperplasia were prospectively randomised in a controlled clinical trial and distributed into two groups. Group A received 0.1% TXA 1000 mg (10 mL) in 1 L of irrigation solution of sterile wash (glycine) during surgery, while Group B received 10 mL distilled water (placebo) in 1 L of irrigation solution of sterile wash (glycine) during surgery. At the end of surgery, a three-way catheter was inserted in the bladder. Group A received local 500 mg of TXA (5 mL), which was dissolved in 100 mL of normal saline solution, while Group B received distilled water (5 mL) dissolved in 100 mL of normal saline solution after which the catheter was clamped. The serum haemoglobin (Hb) concentration, haematocrit (HCT), blood loss volume, Hb concentration in the irrigation fluid, and bladder irrigation volumes were compared between the two groups at three time-points: preoperatively and at 4- and 24-h postoperatively. Coagulation function, complications, thromboembolic events, quality of endoscopic view, surgery duration, and hospital stay were also noted.

**Results:**

Group A had significantly lower blood loss intraoperatively, and at 4- and 24-h postoperatively compared to the control group (*P* < 0.05). The serum Hb concentration, HCT, Hb concentration in the irrigation fluid, and bladder irrigation volumes were significantly lower in the TXA group vs the control group (*P* < 0.001). The shortening of the surgery duration and improvement in the quality of the endoscopic view were significantly noted in the TXA group (*P* = 0.001). However, no thromboembolic events occurred in either group.

**Conclusion:**

The use of TXA in the irrigation fluid during TURP and injection into the bladder postoperatively can reduce blood loss and the need for blood transfusion without increasing the risk of thrombosis.

## Introduction

TURP is one of the most common and well-developed techniques used to treat BPH, recognised as the ‘gold standard’ of the surgical treatments of enlarged prostates [1]. The most relevant complications are the inability to void (5.8%), surgical revision (5.6%), UTI (3.6%), bleeding requiring transfusions (2.9%), and TUR syndrome (1.4%) [2].

As the prostate has a rich blood supply, haemorrhage is one of the most common complications of TURP. Moreover, the development of adenoma is accompanied by a significant increase in angiogenesis and the formation of aberrant blood vessels, which may cause substantial intraoperative bleeding during prostate resection [3].

There are different techniques that can minimise bleeding during endoscopic treatment of BPH such as laser-assisted endoscopic treatment, preoperative prostatic artery embolisation, or pharmacological treatment by antifibrinolytic agents (e.g. tranexamic acid [TXA]).

Blood loss after TURP may be due to an increase in urinary fibrinolytic activity that facilitates the lysis of clots. This rise is due to urokinase release by the prostate. In addition, urine and urothelium contain high concentrations of plasminogen activators that stimulate the fibrinolytic system [4–6]. Therefore, administering antifibrinolytic agents such as TXA may be effective in reducing blood loss during TURP.

Locally administering TXA has been shown to reduce blood loss effectively in some surgical fields such as cardiac surgery, oral surgery, dental surgery, knee arthroplasty and endoscopic sinus surgery, without causing any significant risk, systemic absorption, or thromboembolic disorders [7,8].

There are few studies that have assessed the local use of TXA in the field of urology. Pourfakhr et al. [9] reported that the local administration of TXA during open prostatectomy significantly reduced blood loss. Also, Bansal and Arora [10] assessed the use of TXA locally in percutaneous nephrolithotomy (PCNL) and reported that TXA in the irrigant is safe and significantly reduces perioperative blood loss and the need for blood transfusion. To our knowledge, the role of TXA in the irrigation fluid during TURP has never been studied.

Thus, we conducted the present study to evaluate the safety and efficacy of administering TXA locally for patients undergoing TURP. The primary endpoint was to observe a reduction in blood loss while the secondary endpoint was to evaluate the adverse effects related to the use of TXA.

## Patients and methods

The Ethics Committee of Ain Shams University approved the study (FWA00017585). All patients included in this study were given a written informed consent after being provided with an explanation of the study procedures and the follow-up course.

Sample size was calculated using the PASS 11 program, setting the type-1 error (α) at 0.05 and power at 100%. Results from a previous study showed that the mean (SD) blood loss in the treatment group (TXA) was 64 (5.2) vs 77.5 (5) mL in the control group [11]. Based on this, we determined that a sample size of 25 patients per group (50 total) would be needed.

From March 2020 to May 2021, a prospective, randomised clinical study of 50 patients with BPH (aged 50–85 years) with a prostate weight of 50–80 g undergoing TURP were randomly distributed by the closed envelope method into two groups: Group A, representing the TXA group and Group B representing the control group. The study took place at the Faculty of Medicine in Ain Shams University. Exclusion criteria included patients hypersensitive to TXA, or on antiplatelet and anticoagulant drugs, or who had a history of thrombotic events, bleeding disorders, chronic kidney disease, abnormal liver function test, cardiovascular disease treated with a drug-eluting stent, bladder stone, urethral stricture, or who had had previous prostate surgery, prostate cancer, had a UTI or who had received 5α-reductase inhibitors.

All patients were tested for hypersensitivity to TXA by being given an intradermal injection of 0.5 mL drug with a 1:1 dilution in normal saline. The test was considered positive if a wheal of >1 cm in diameter was observed after 20 min at the site of injection [10].

**Figure 1.**
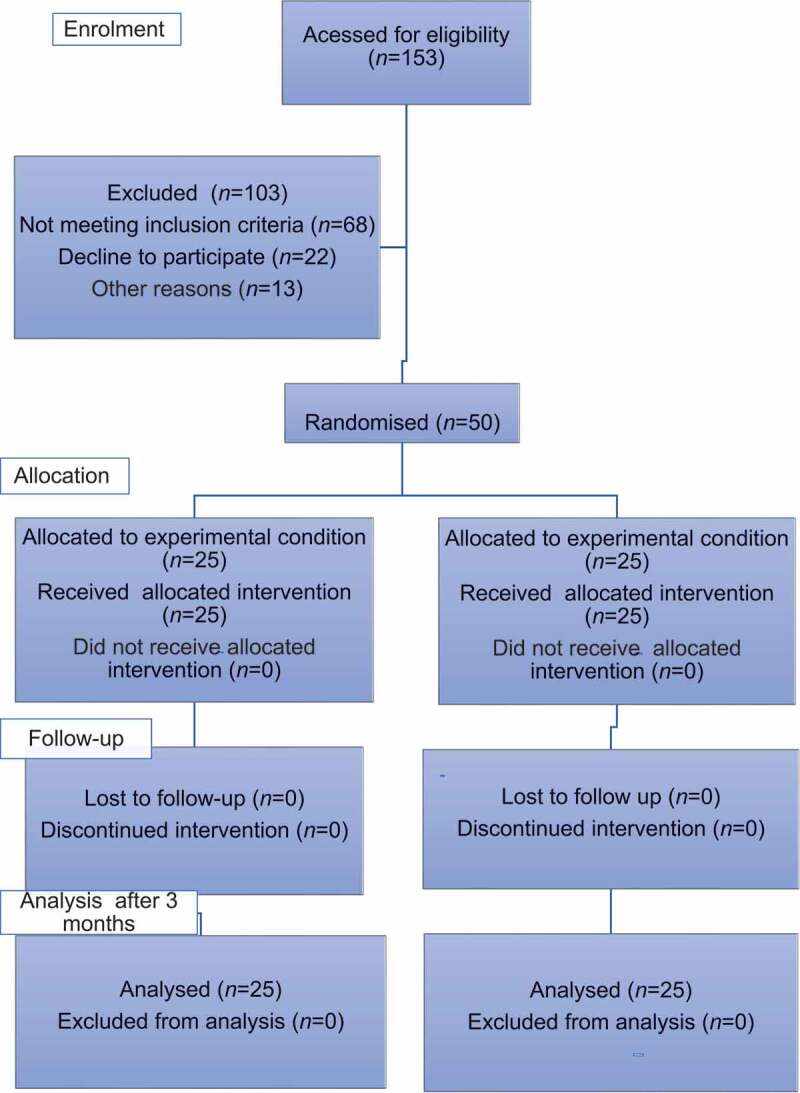
Consolidated Standards of Reporting Trials (CONSORT) flow chart.

A total of 153 patients with urinary symptoms because of BPH were assessed for eligibility in the study. A total of 103 patients were excluded for different reasons: 68 for not meeting inclusion criteria, 22 for refusal to participate, and 13 for other reasons. The remaining 50 patients were randomly divided into two equal groups by using the closed envelope method (Figure 1). Group A (TXA group) received 0.1% TXA solution (1000 mg with total volume of 10 mL) in 1 L of irrigation solution of sterile wash (glycine). Group B (control group) received 10 mL of distilled water (placebo) in 1 L of irrigation solution sterile wash (glycine) [10]. At the end of the surgery, a three-way catheter was inserted in the bladder, where Group A received 500 mg TXA with a total volume of 5 mL dissolved in 100 mL normal saline solution, while in Group B, distilled water (5 mL) dissolved in 100 mL of normal saline solution was used. In both groups, urinary flow was closed for 15 min by clamping the catheter after which bladder-washing was continued [12].

Operative procedure: the operation were performed by the same expert surgeon. Prophylactic antibiotics were administrated during anaesthesia. After spinal anaesthesia, the patient was placed in lithotomy position and TURP was done using a 26-F continuous flow resectoscope. Finally, a 22-F three-way catheter was placed, and postoperative irrigation started until the wash became clear, then the catheter was removed.

Outcome measure: we compared the two groups with regards to age, prostate size, IPSS, duration of the procedure (from the start of resection until the application of the urethral catheter), postoperative hospital stay length, quality of endoscopic view (using a scale of 3; where 1 = good, 2 = fair, and 3 = poor), and intra- and postoperative blood loss. Blood loss was measured from bladder irrigation fluid, which was collected at three time-points: immediately after the surgery (T1), 4 h after TURP (T2), and 24 h after TURP (T3) [11]. Heparin (1000 U) was added to the collection buckets to prevent coagulation of the irrigation solution. The 5-mL irrigation fluid samples were transferred into an EDTA vial for haemoglobin (Hb) estimation. The HemoCue method was used to calculate the amount of blood loss [13]. Within this method, the Hb (g/dL) value of the irrigation fluid was multiplied by the volume of total irrigation fluid (mL) used. The result was divided by the preoperative value of serum Hb (g/dL), and thus, the total amount of blood loss (dL) was obtained, then converted to millilitres. A decrease was noted in in Hb and haematocrit (HCT) (serum Hb and HCT levels were measured preoperatively and at 4- and 24-h postoperatively) and blood transfusion rates. Coagulation functions including prothrombin and activated partial thromboplastin time were measured in the two groups preoperatively and at 4-h postoperatively. We carefully monitored the patients for level of consciousness, lower limb oedema, breathing status, chest tightness, as well as urine output after TURP to exclude the side-effects of TXA such as myocardial infarction, pulmonary embolism, seizures, and renal failure.

Statistical analysis: we use the IBM Statistical Package for the Social Sciences (SPSS®) version 23 (IBM Corp., Armonk, NY, USA). Continuous variables are expressed as mean and standard deviation (SD). Categorical variables are expressed as frequencies and percentages. The Student’s *t*-test was used to assess the statistical significance of the difference between the two study groups’ means. Fisher’s exact test was used to examine the relationship between categorical variables. A significance level of *P* < 0.05 was used in all tests.

## Results

For the preoperative data and IPSS there were no statistically significant differences between the two groups, as seen in Table 1. On the other hand, there were statistically significant differences between the two groups for Hb and HCT at 4- and 24-h postoperatively.

**Table 1.** Comparison between the two groups regarding preoperative data

Variable	Control group (*N* = 25)	TXA group (*N* = 25)	*P*
Age, years, mean (SD, range)	68.88 (7.82, 57–80)	69.36 (7.88, 57–80)	0.830
Prostate size, g, mean (SD, range)	68.88 (9.27, 50–80)	70.28 (9.17, 50–80)	0.594
Preoperative PVR, mL, median (IQR)	320 (210–500)	325 (240–500)	0.879
Postoperative PVR, mL, median (IQR)	0	0	
Total PSA, ng/mL, mean (SD, range)	6.15 (1.2, 4–10)	5.57 (1.04, 3–7.2)	0.074
Free PSA, ng/mL, mean (SD, range)	1.42 (0.34, 1–2.5)	1.58 (0.33, 1–2.2)	0.106
Preoperative IPSS, mean (SD, range)	26.96 (4.16, 19–35)	25.84 (3.59, 19–33)	0.313
Postoperative IPSS, mean (SD, range)	7.96 (2.46, 4–14)	8.12 (2.39, 4–14)	0.816
**Preoperative**			
Hb, g/dL, mean (SD, range)	13.70 (0.77, 12–15)	14.11 (0.82, 12–16)	0.077
HCT, %, mean (SD, range)	42.96 (1.88, 40–47)	42.88 (2.22, 39–27)	0.891
**4-h postoperatively**			
Hb, g/dL, mean (SD, range)	12.6 (0.82, 10.6–14.1)	13.24 (0.77, 11.5–15)	0.006
HCT, %, mean (SD, range)	34.68 (1.52, 32–38)	36.96 (1.37, 34–39)	<0.001
**24-h postoperatively**			
Hb, g/dL, mean (SD, range)	12.33 (0.85, 10.3–13.8)	13.01 (0.78, 11.2–14.8)	0.005
HCT, %, mean (SD, range)	32.48 (1.5, 31–37)	34.8 (1.38, 32–37)	<0.001

PVR, post-void residual urine volume.

Blood loss was reduced significantly in the TXA group vs the control group at the three different time-points (T1, T2, T3), and there was a significant difference between the two groups in the total blood loss, as shown in Table 2. For the Hb concentration in the irrigation fluid, there was a significant reduction in the Hb concentration of the irrigation fluid at T1 and T2, but without a significant difference at T3 (Table 2). Irrigation fluid volumes revealed a significant difference between the two group at all three time-points (T1, T2, T3), reflecting a significant statistical difference in total irrigation volumes between the two groups (Table 2)

**Table 2.** Comparison between the two study groups for blood loss, Hb concentration in the irrigation fluid and the irrigation volume at the three time-points (T1, T2, T3)

	Control group (*N* = 25)	TXA group (*N* = 25)	*P*
Blood loss, mL, mean (SD, range)			
T1	500.28 (96.69, 346–777)	395.44 (109.73, 286–740)	0.001
T2	56.6 (12.66, 34–90)	36.6 (15.74, 16–93)	<0.001
T3	87.28 (17.17, 51–126)	51.4 (16.89, 20–83)	<0.001
Total	644.16 (101.17, 478–924)	483.44 (113.49, 360–815)	<0.001
Irrigation fluid Hb concentration, g/dL, mean (SD, range)			
T1	0.26 (0.05, 0.2–0.4)	0.23 (0.06, 0.16–0.37)	0.018
T2	0.15 (0.02, 0.12–0.19)	0.12 (0.05, 0.04–0.28)	0.005
T3	0.07 (0.02, 0.02–0.1)	0.06 (0.02, 0.02–0.09)	0.705
Total	0.48 (0.06, 0.4–0.64)	0.41 (0.09, 0.28–0.6)	0.003
Irrigation volume, mL, mean (SD, range)			
T1	2530.20 (130.22, 2200–2700)	2420.80 (130.85, 2200–2800)	0.009
T2	510 (80.78, 350–650)	430.16 (100.69, 300–700)	0.007
T3	1470.00 (110.90, 1200–1650)	1380.20 (140.13, 1000–1600)	0.021
Total	4510.20 (200.93, 4050–4850)	4240.16 (270.35, 3700–4750)	<0.001

A significant statistical difference was noted between the two groups for the surgery duration and quality of the endoscopic view (Table 3). However, there was no statistically significant difference between both groups for perioperative complications, bleeding profile, and length of hospital stay (Table 3).

**Table 3.** Comparison between the two groups for complications, bleeding profile, surgery duration, hospital stay and quality of endoscopic view

Variable	Control group (*N* = 25)	TXA group (*N* = 25)	*P*
Complications, *n* (%) No TUR syndrome	24 (96.0) 1 (4.0)	25 (100.0) 0	0.312
INR, mean (SD)	1.03 (0.06)	1.02 (0.04)	0.427
Surgery duration, min, mean (SD)	99.2 (8.74, 80–120)	91.4 (6.85, 80–100)	0.001
Hospital stay, days, mean (SD)	2.12 (0.33, 2–3)	2.04 (0.2, 2–3)	0.307
Quality of endoscopic view, *n* (%) Poor Fair Good	5 (20.0) 15 (60.0) 5 (20.0)	3 (12.0) 8 (32.0) 14 (56.0)	0.032

INR, international normalised ratio.

## Discussion

The intravenous use of the TXA poses some risks such as thrombosis and pulmonary embolism [14]. To avoid these systemic side-effects, the topical use of TXA during surgeries should be evaluated.

A Cochrane review included 29 trials, involving 2612 participants who underwent total hip and knee arthroplasty, spine surgery, cardiac surgery, and dental surgery, and reported that the administration of local TXA reduced blood loss by 29% [15].

To our knowledge, there are few reported studies about the use of TXA either intravenously or topically in urosurgery such as in PCNL. Kumar et al. [16] used TXA intravenously in PCNL and observed that the Hb drop was 1.39 g/dL in patients receiving TXA, compared with 2.31 g/dL in the control group (*P* < 0.001). Bansal and Arora [10] used 0.1% TXA in the irrigation fluid during PCNL and reported that there was a significant decrease in perioperative blood loss compared with placebo (154.5 vs 212.6 mL, *P* < 0.001); the mean drop in Hb and HCT in the TXA group was significantly lower than in the placebo group (1.71 vs. 2.67 g/dL, *P* < 0.001; 4.23 vs 7.78, *P* < 0.001) respectively. Also, the mean operative time of the TXA group was significantly lower compared to placebo (68.45 vs. 87.62 min, *P* < 0.001) [10]. Also, Samir et al. [17] reported that high-dose TXA was effective in controlling blood loss during bipolar TURP in patients with large prostates. However, no study has been conducted on the use of TXA in the irrigation fluid during TURP.

In the present study, we found that the use of TXA in the irrigation fluid significantly decreased blood loss. This was in agreement with Pourfakhr et al. [9], who reported that the local administration of TXA (500 mg TXA with 5 mL total volume) after prostatectomy significantly reduced blood loss. The intervention group had a mean blood loss of 340 mL and the control group had a mean blood loss of 515 mL, which was statistically significant (*P* = 0.01). Moreover, the decreases in Hb and HCT levels after surgery were statistically significant (*P* = 0.04 and *P* = 0.05, respectively). On the other hand, Moharamzadeh et al. [12] reported that there was no significant effect of the topical use of TXA on blood loss. The study was conducted on 50 patients complaining of painless haematuria. During bladder irrigation, TXA or placebo solutions were injected into the bladder via a Foley catheter. That result may be due to the small sample size of the study, as well as the fact that the source of haematuria could be from the upper tract, which is not necessarily affected by bladder wash with TXA.

Regarding the drop in Hb concentration, our present results show that there was a significant reduction in the total serum Hb concentration in the TXA group (mean [SD] 1.10 [0.24] g/dL) vs the control group (mean [SD] 1.37 [0.31] g/dL). This is in line with Pourfakhr et al. [9], in which the local administration of TXA after prostatectomy was significantly effective in preventing postoperative Hb reduction (*P* = 0.04). Also, Karkhanei et al. [18] reported that the amount of Hb reduction in the control group, at 1.22 g/dL, was 0.93 higher than that of the case group (*P* < 0.05) when intravenous TXA was used during TURP. On the other hand, Moharamzadeh et al. [12] reported no significant effect from the topical use of TXA on the reduction of total Hb concentration. The Hb level at 24 h. after the intervention in the TXA group vs the control group was recorded at a mean (SD) of 10.00 (1.70) vs 9.56 (1.87) g/dL (*P* = 0.39). Also, Jendoubi et al. [19] reported that there was no significant effect from the intravenous use of TXA on the reduction of total Hb concentration during bipolar TURP (mean [SD] 1.37 [0.69] vs 1.72 [1.23] g/dL, *P* = 0.256). This could be explained by the better haemostatic capacity in the bipolar current, which helps to reduce perioperative bleeding.

Additionally, there was a significant difference between the two groups’ serum HCT at both 4- and 24-h postoperatively. The Pourfakhr et al. [9] study agrees with our present study, as they reported that a local administration of TXA significantly reduced postoperative HCT loss (*P* = 0.05).

Finally, with regards to the Hb concentration in the irrigation fluid, the present study reports that local TXA significantly reduced the Hb concentration in the irrigation fluid and irrigation fluid volume (*P* = 0.003 and *P* < 0.01, respectively). This is in agreement with Vezhaventhan et al. [20], who reported that the Hb loss in the irrigating fluid was significantly lower in the group of patients given intravenous TXA than in the control group (*P* < 0.01); also, the volume of irrigation fluid during TURP surgery was 16.5 L in the TXA group vs 18.48 L in the non-tranexamic group (*P* < 0.001).

The present study reports that local TXA significantly reduces surgery duration and improves the endoscopic view during surgery. Karkhanei et al. [18] agreed with these data results when they reported that intravenous TXA use results in a shorter operation time. The mean (SD) time of operation was 53.57 (16.43) vs 120.71 (47.76) min in the case and control group, respectively (*P* < 0.05). They also reported that intravenous TXA resulted in better vision and surgeon satisfaction during the operation. The frequency of high satisfaction in the case and control groups was (74.3% and 8.65%), and low satisfaction was 0% and 42.69%, respectively (*P* < 0.05).

We have some limitations in the present study: small sample size and the need for external validation in multicentre studies. Also, we did not rely on venous lower limb doppler to exclude asymptomatic deep vein thrombosis.

## Conclusion

The local use of TXA in the irrigation fluid during TURP surgery and postoperatively could be used as an option to reduce blood loss without the risk of thrombosis.

## Abbreviations

HCT: Haematocrit; Hb: Haemoglobin; PCNL: Percutaneous Nephrolithotomy; TXA: Tranexamic Acid
